# A generalised computer vision model for improved glaucoma screening using fundus images

**DOI:** 10.1038/s41433-024-03388-4

**Published:** 2024-11-05

**Authors:** Abadh K. Chaurasia, Guei-Sheung Liu, Connor J. Greatbatch, Puya Gharahkhani, Jamie E. Craig, David A. Mackey, Stuart MacGregor, Alex W. Hewitt

**Affiliations:** 1 Menzies Institute for Medical Research, University of Tasmania, Hobart, TAS Australia; 2 Centre for Eye Research Australia, Royal Victorian Eye and Ear Hospital, East Melbourne, VIC Australia; 3 Ophthalmology, Department of Surgery, University of Melbourne, East Melbourne, VIC Australia; 4 QIMR Berghofer Medical Research Institute, Brisbane, QLD Australia; 5 School of Medicine, University of Queensland, Brisbane, QLD Australia; 6 Faculty of Health, School of Biomedical Sciences, Queensland University of Technology, Brisbane, QLD Australia; 7 Department of Ophthalmology, Flinders University, Flinders Medical Centre, Bedford Park, SA Australia; 8 Lions Eye Institute, Centre for Vision Sciences, University of Western Australia, Perth, WA Australia

**Keywords:** Optic nerve diseases, Outcomes research

## Abstract

**Importance:**

Worldwide, glaucoma is a leading cause of irreversible blindness. Timely detection is paramount yet challenging, particularly in resource-limited settings. A novel, computer vision-based model for glaucoma screening using fundus images could enhance early and accurate disease detection.

**Objective:**

To develop and validate a generalised deep-learning-based algorithm for screening glaucoma using fundus image.

**Design, setting and participants:**

The glaucomatous fundus data were collected from 20 publicly accessible databases worldwide, resulting in 18,468 images from multiple clinical settings, of which 10,900 were classified as healthy and 7568 as glaucoma. All the data were evaluated and downsized to fit the model’s input requirements. The potential model was selected from 20 pre-trained models and trained on the whole dataset except Drishti-GS. The best-performing model was further trained to classify healthy and glaucomatous fundus images using Fastai and PyTorch libraries.

**Main outcomes and measures:**

The model’s performance was compared against the actual class using the area under the receiver operating characteristic (AUROC), sensitivity, specificity, accuracy, precision and the F1-score.

**Results:**

The high discriminative ability of the best-performing model was evaluated on a dataset comprising 1364 glaucomatous discs and 2047 healthy discs. The model reflected robust performance metrics, with an AUROC of 0.9920 (95% CI: 0.9920–0.9921) for both the glaucoma and healthy classes. The sensitivity, specificity, accuracy, precision, recall and F1-scores were consistently higher than 0.9530 for both classes. The model performed well on an external validation set of the Drishti-GS dataset, with an AUROC of 0.8751 and an accuracy of 0.8713.

**Conclusions and relevance:**

This study demonstrated the high efficacy of our classification model in distinguishing between glaucomatous and healthy discs. However, the model’s accuracy slightly dropped when evaluated on unseen data, indicating potential inconsistencies among the datasets—the model needs to be refined and validated on larger, more diverse datasets to ensure reliability and generalisability. Despite this, our model can be utilised for screening glaucoma at the population level.

## Introduction

Glaucoma is a multifactorial optic neuropathy that affects millions of people worldwide [1]. It is characterised by progressive degeneration of the optic nerve head (ONH), leading to irreversible vision loss if left undiagnosed and untreated in a timely manner [2, 3]. The early stages of the disease are usually asymptomatic, thus, proactive and effective screening methods are essential for preventing substantial vision loss. However, present glaucoma detection methods are restricted by improved access to comprehensive eye care, particularly in low- and middle-income countries where access to advanced diagnostic equipment and trained glaucoma specialists is limited [4, 5]. Moreover, current screening methods have a significant false-positive rate, which can cause unnecessary anxiety and burden [6, 7]. These challenges highlight the need for innovative approaches in glaucoma screening, including advancements in artificial intelligence.

Traditional techniques for glaucoma detection typically include measuring intraocular pressure (IOP), assessing the ONH using optical coherence tomography (OCT) and performing visual field tests. However, these methods can be time-consuming, expensive and prone to variability [8]. Measurement of IOP, while useful, often misses cases of normal-tension glaucoma where optic neuropathy occurs despite statistically normal pressure levels [9]. Furthermore, these clinical tests require interpretation by an experienced clinician, introducing subjective bias and limiting accessibility in resource-constrained settings. Although conventional diagnostic procedures are valuable, they serve as a ground for utilising more advanced technologies. Further, the need for in-person testing limits accessibility for patients in remote or underserved areas. These challenges underscore the need for more effective, accessible and unbiased approaches to screen glaucoma.

Computer vision-based models to diagnose glaucoma using fundus images [10]—a widely used and non-invasive imaging technique that can provide valuable details about ONH conditions—have shown promise. Fundus images reveal the early signs of glaucoma, which are relevant for screening and managing glaucoma [11, 12]. This imaging modality can be integrated with deep-learning algorithms to automatically extract glaucomatous features from fundus images. These algorithms have the potential to enhance the efficiency of glaucoma screening and diagnosis [13]. Nevertheless, most existing studies focus on specific populations or regions with limited data that lack the potential to be a generalisable model for glaucoma screening [14–17].

In this study, we sought to develop and validate a generalised deep-learning-based algorithm from 20 publicly accessible glaucomatous fundus data using the top-performing model out of 20 pre-trained models to ensure an accurate screening model for the disease, making it more useful in ophthalmic practice. This algorithm may provide a practical and cost-efficient way for screening glaucoma at the population level.

## Methods

### Description of datasets

We used 20 publicly accessible glaucoma datasets from cohorts across the world (eFig. 1 in *Supplementary*) [18, 19]. A full description of the cohorts is contained in the Supplementary Text. In brief, these datasets involved people from diverse ethnic groups, with retinal images being obtained using various cameras at differing resolutions. Our study design depends on pre-existing and anonymised data. Nevertheless, all investigations followed relevant guidelines and regulations, ensuring high ethical compliance standards throughout the study. All the datasets, excluding the Drishti-GS cohort, were used to train the classification model. The Drishti-GS dataset was used for external validation of our model [20]. The dataset’s ground truth was constructed by four experts with varying clinical experience (3–20 years), making it an ideal option for external validation.

### Data pre-processing and augmentation

We utilised a diverse set of fundus images from 20 publicly accessible databases. These databases exhibited considerable variability in fundus images; some datasets had only ONH disc images, while others contained complete retinal fundus images. We pre-processed different image types to create a uniform set of ONH disc images—using OpenCV to identify and isolate circular objects (ONH) in fundus images [21]. This involved converting to grayscale, applying a Gaussian blur and using the Hough Circle Transform to improve circle detection [22]. The ONH images were then automatically cropped and resized to a uniform resolution of 512 × 512. If any image did not detect the ONH or if the ONH was off-centre, the image was manually cropped, focusing on the most clinically significant aspects of the fundus images [23]. After cropping, the image was downsized to 224 × 224 with three channels to ensure consistent dimensions for input into our deep-learning models for glaucoma screening.

Acknowledging the constraints of limited high-quality, publicly accessible fundus images, we increased our training dataset via data augmentation—a technique pivotal to preventing model overfitting and enabling robustness and generalisability [24]. To enhance our dataset’s diversity, various data augmentation techniques were employed, including random rotation, cropping, flipping, scaling, lighting adjustment, affine transformations and zoom, coupled with normalisation using ImageNet statistics, as shown in eTable 1 in *Supplementary*. Importantly, these augmentation techniques undistorted the critical features of the ONH disc images relevant for glaucoma diagnosis.

### Model selection and training

Our study compared 20 deep-learning architectures for classifying healthy and glaucomatous disc images. This experiment included a variety of models, including ResNets (18, 34, 50, 101, 152 layers), VGG (16, 19 layers with batch normalisation), AlexNet, DenseNets (121, 161, 169, 201 layers), SqueezeNets (1.0, 1.1), GoogLeNet, ShuffleNet, ResNext (50_32x4d, 101_32x8d) and Wide ResNets (50_2, 101_2). We utilised the Fastai library using the cnn_learner function to create a learner object combining model, data, loss functions, and class weights [25]. All the models were trained for three epochs using a fine-tuning approach, and then we evaluated the accuracy of each model on our validation set, which consisted of 20% of the total 18,366 images. The process was repeated three times using randomly split data for training and testing purposes. For the comparative performance of the different models, we plotted a bar chart of the accuracies in eFig. 2 in *Supplementary*. The best-performing model (vgg19_bn using pre-trained weights from ImageNet [26]) was selected based on its performance and complexity. The vgg19_bn incorporates batch normalisation, which accelerates training and increases model stability by reducing internal covariate shifts [27]. The model’s parameters were altered to better fit our dataset by fine-tuning it for 15 epochs until the validation loss stopped decreasing. Following fine-tuning, we used the one-cycle policy to train the model, which adjusts the learning rate and momentum for more efficient training [28]. The training was terminated when the validation loss failed to improve over two consecutive epochs, enabled by a callback function.

The degree of agreement with the established ground truth of publicly available datasets varied considerably across datasets [29]. Subsequently, we employed the ImageClassifierCleaner from the Fastai library to review and clean our dataset [30]—excluding around 1% of the total images the model had incorrectly predicted. We repeated the shuffling and training process seven times and removed ~7% of the images to improve the overall quality of the dataset. The data-cleaning process effectively reduced the number of misclassified images from the dataset. The clean dataset showed a marked classification imbalance between healthy and glaucomatous fundus images: 59.7% healthy and 40.3% glaucomatous. We employed a weighted cross-entropy, a loss function commonly utilised in training a classification model, to address the problem of imbalanced data [31]. The model was re-trained on this refined data, which is more consistent and less likely to mislead the model. Finally, we evaluated the model’s performance on unseen data.

### Model decision visualisation

We employed Gradient-weighted Class Activation Mapping (Grad-CAM) to improve the interpretability and transparency of deep-learning models—Grad-CAM provides valuable insights into the decision-making process by highlighting the important regions in an image relevant to predicting the healthy and glaucomatous disc images [32]. Grad-CAM calculates the gradient of the image score for a particular class and estimates the gradient of the final classification score concerning the weights of the last convolutional layer. These visualisations help uncover the underlying patterns and features that influence the model’s decision for clinicians, making it easier to validate and understand its predictions.

### Statistical analyses

To evaluate the performance of our deep-learning model in distinguishing between healthy and glaucomatous discs, we applied various evaluation metrics: the AUROC, sensitivity (or recall), specificity, accuracy, precision and the F1-score. These metrics provide a comprehensive measure of the model’s accuracy, ability to avoid false positives and balance between precision and recall. The model’s prediction probabilities and true labels produced by the model were transformed into numpy arrays to facilitate subsequent computations. To validate the performance of these measurements, we utilised a bootstrap resampling method [33]. This method involved 4000 iterations of resampling with replacement from our dataset, which allowed for calculating 95% confidence intervals for each performance metric.

The experiment was performed on a virtual Ubuntu desktop (version 22.04) using NVIDIA A100 with 40GB of GPU RAM at Nectar Research Cloud [34]. Python (version 3.10.6) along with PyTorch (version 2.0.0+cu117), Fastai (version 2.7.12), TorchVision (version 0.15.1+cu117), Matplotlib (version 3.5.1) and Scikit-learn (version 1.2.2) libraries were employed [35–37].

## Results

### Data description

A total of 117,152 fundus images were retrieved from 20 databases with 12 unique countries and two unknown countries (Table 1). Non-referral glaucomatous images were excluded from the EyePACS dataset to prevent bias towards the healthy group, and 512 ungradable images were also excluded from the datasets. Our deep-learning model was trained and evaluated on an extensive dataset of 18,468 disc images from multiple clinical settings worldwide; 10,900 images were healthy, while 7568 had glaucoma.

**Table 1:** Overview of the dataset sources and distribution of fundus images across classes.

Dataset	Country of data origin	Glaucoma	Healthy	Ungradable
Training Dataset				
HRF [44]	Germany and the Czech Republic	15	15	0
ACRIMA [45]	Spain	396	309	0
REFUGE1 [46]	China	120	1080	0
RIGA [47]	Saudi Arabia and France	56	693	0
RIM-ONE-DL [48]	Spain	172	313	0
Retina (sjchoi86-HRF) [49]	Unknown	101	300	0
DRIONS-DB [50]	Spain	15	95	0
ODIR [51]	China	200	2427	389
ORIGA [52]	Singapore	168	482	0
LAG [53]	China	1607	3139	108
JSIEC [54]	China	13	38	0
BIOMISA [55]	Pakistan	17	22	0
BEH [56]	Bangladesh	171	463	0
VEIRC [57]	India	32	225	0
LES-AV [58]	Unknown	11	11	0
G1020 [59]	Germany	290	715	15
PAPILA [60]	Spain	88	333	0
KEH (Harvard) [61]	Korea	756	209	0
EyePACS [62]	USA	3270	98,172^a^	0
External Validation				
Drishti-GS1 [63]	India	70	31	0
Total (117,152)		7568	109,072	512

^a^Excluded from the training set.

### Model performance

Our best-performing model demonstrated robust performance in discriminating between healthy and glaucomatous disc images, as shown in Table 2. The model achieved high accuracy, precision, recall and specificity in classifying the images. It successfully maintained a balance between sensitivity and specificity, as indicated by the AUROC of 0.9920, showing a strong ability to accurately identify glaucoma and healthy discs. Moreover, the F1 scores, which consider both precision and recall, were higher than 96% for both classes.

**Table 2:** Key performance metrics of the classification model.

Classification Metrics	Glaucoma (*n* = 1381) Mean, [95% CI]	Healthy (*n* = 2030) Mean, [95% CI]
AUROC	0.9920, [0.9920, 0.9921]	0.9920, [0.9920, 0.9921]
Accuracy	0.9671, [0.9670, 0.9672]	0.9671, [0.9671, 0.9672]
Sensitivity (Recall)	0.9530, [0.9528, 0.9532]	0.9768, [0.9767, 0.9769]
Specificity	0.9768, [0.9767, 0.9769]	0.9530, [0.9529, 0.9532]
Precision	0.9654, [0.9652, 0.9656]	0.9683, [0.9682, 0.9684]
F1-score	0.9592, [0.9591, 0.9593]	0.9725, [0.9724, 0.9725]

*CI* confidence intervals.

### Generalisability of the model

**Fig. 1:**
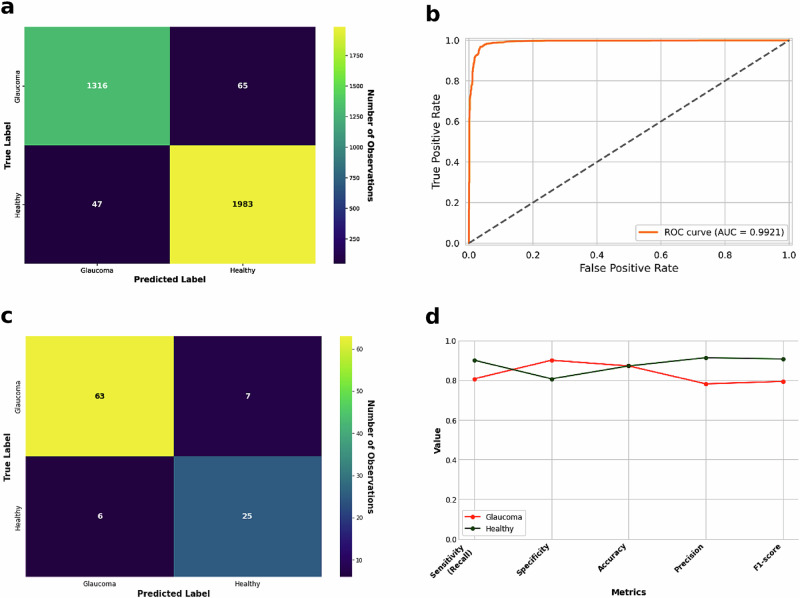
A visual representation of the model’s performance. **a** Displays the confusion matrix from the validation data. **b** Illustrates the AUROC curve on the validation set, reflecting the model’s discriminative power between healthy and glaucomatous discs. For external validation on the Drishti dataset, (**c**) presents another confusion matrix showcasing the model’s predictive performance, while (**d**) features a plot summarising all key performance metrics such as sensitivity, specificity, accuracy, precision and F1-score.

To ensure the applicability of our model across populations, we validated it on the DrishtiI-GS dataset, which was excluded from the model’s training and validation steps. The model demonstrated significant generalisability, as highlighted by the performance metrics in Fig. 1. The model attained an overall accuracy of 87.13% (0.8713) and AUROC of 87.51% (0.8751) for distinguishing between healthy and glaucomatous discs. This performance on the DRISHTI-GS dataset suggests our best-performing model (vgg19_bn) has potential for glaucoma screening. However, further testing and validation on multiple datasets are essential to confirm its generalisability and reliability across different populations.

### Model errors: insights from the top losses

**Fig. 2:**
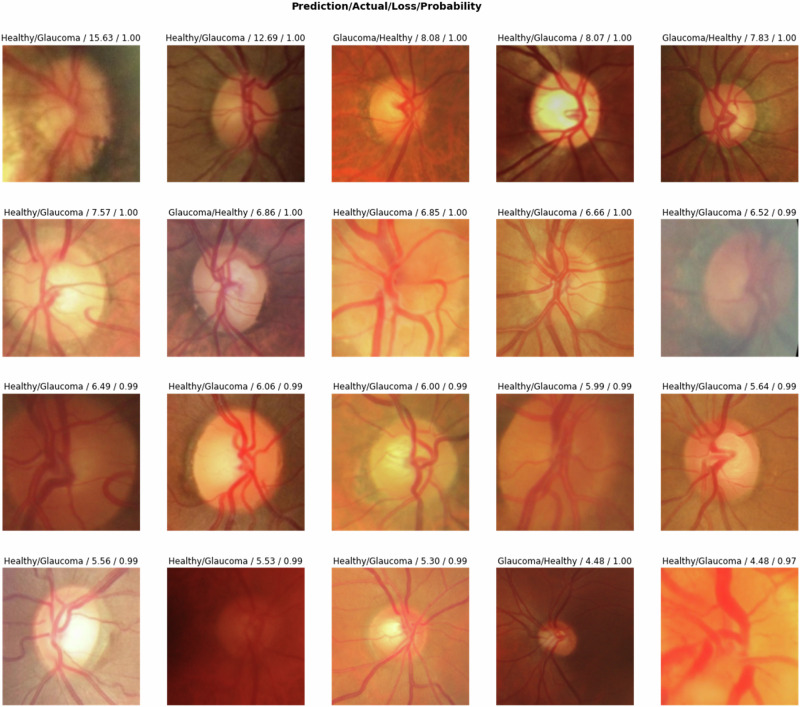
Visualisation of top losses. The model predicted the top 20 fundus images associated with the greatest prediction loss, indicating where the model’s predictions were furthest from the actual classes.

Our model for glaucoma screening had made some incorrect predictions on the validation set; 47 healthy images were wrongly categorised as having glaucoma (false positives), and 65 actual cases of glaucoma were missed (false negatives) (Fig. 1). Examination of the 47 misclassified healthy images revealed certain features, such as optic disc cupping, which could be associated with glaucoma. Among the 65 actual glaucoma cases the model missed, several could be early or borderline cases where pathological changes in the optic disc are less distinct. The conclusions from this error analysis provide valuable insights into areas for improving our model. To further understand the nature of our model’s misclassifications, we utilised the top_losses function from the Fastai library to visualise the instances with the highest loss, shown in Fig. 2. This analysis provides a comprehensive picture of our model’s strengths and weaknesses, guiding us in refining our model for improved accuracy in glaucoma screening.

### Gradient-weighted class activation mapping (Grad-CAM)

**Fig. 3:**
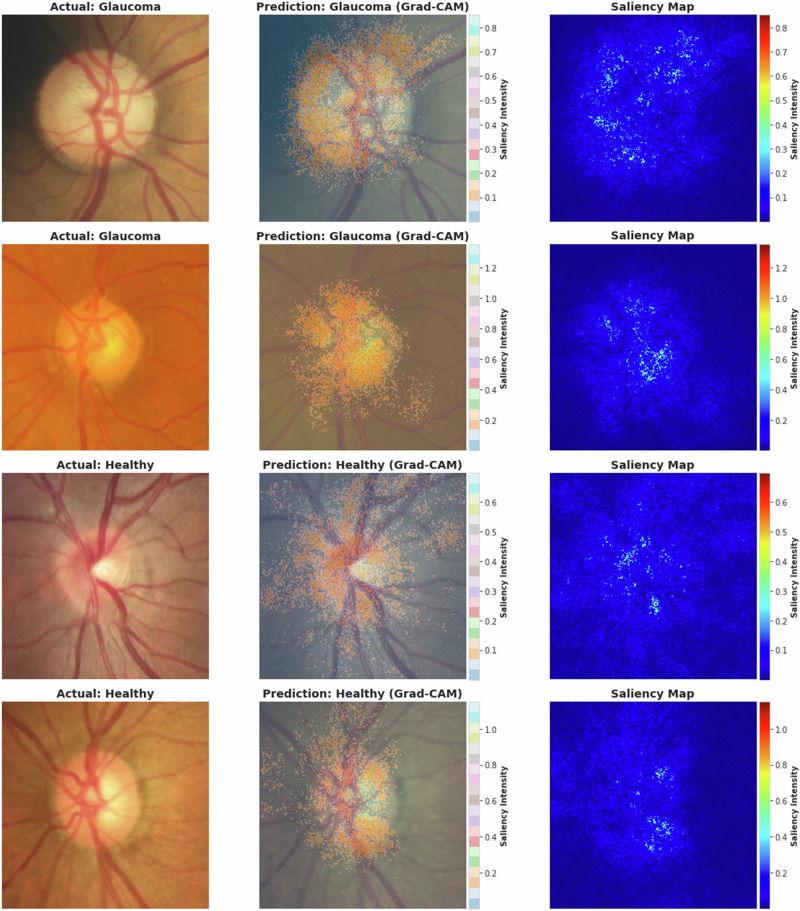
The Grad-CAM saliency maps for glaucoma classification. The saliency map (right) and its overlay on the original image (middle) highlight regions influencing the model’s predictions for each image.

Our classification model achieved promising results, exhibiting a robust capacity to screen glaucoma at a population level using fundus images. However, to further evaluate the decision-making process of our deep-learning model, we utilised Grad-CAM to visualise which regions in the fundus images influenced the model’s classification results. Grad-CAM heatmaps helped visualise the critical areas of fundus images that the model focused on to discriminate between healthy and glaucomatous discs. The heatmaps, represented in terms of higher-intensity-coloured regions superimposed on the fundus images, provided an intuitive understanding of the salient features recognised by our model. Moreover, localised areas around the optic nerve and retinal nerve fibre layer, where early signs of glaucomatous damage commonly occur, were also emphasised in the heatmaps (Fig. 3).

The Grad-CAM visualisations provided valuable insights into classifying fundus images as healthy or glaucomatous. As expected, in glaucomatous images, the heatmaps frequently highlighted the ONH, a critical region for diagnosing glaucoma due to characteristic features such as increased cup-to-disc ratio and neuroretinal rim thinning—which suggests the model correctly focused on clinically relevant areas when detecting glaucoma. Interestingly, healthy images demonstrated a diffused heatmap, implying that the model’s decision was guided more by the absence of pathological traits than the presence of specific healthy features. Saliency maps are primarily clustered at the ONH of glaucomatous discs.

## Discussion

In this study, we aimed to develop and validate a generalised glaucoma screening model. The best-performing model (vgg19_bn) achieved promising accuracy, highlighting the potential of our approach to revolutionise the landscape of glaucoma screening. To the best of our understanding, no prior study had attempted to use extensive glaucomatous fundus data from diverse demographics and ethnicities, with images from various fundus cameras with different resolutions. Most deep-learning-based models were trained on a limited dataset for classifying healthy and glaucomatous fundus images from a single institution, which made the models non-generalisable for different populations and settings [10]. Our training dataset included 7,498 glaucoma cases and 10,869 healthy cases, gathered from 19 different datasets. This dataset—one of the most extensive clusters of fundus images ever used to develop a generalised glaucoma screening model—represents a wide range of ethnic groups and fundus cameras used, which could improve our model’s performance and make it more applicable globally. The best-performing model was selected in this study out of 20 pre-trained models (eFig. 2 in *Supplementary*)—choosing the right deep-learning architecture for a specific task is extremely important [38]. This extensive and diverse dataset using the potential deep-learning architecture can enhance the model’s generalisability, making it a versatile and practical tool for glaucoma screening in diverse populations.

Our best-performing model exhibited exceptional discriminative ability between glaucomatous and healthy discs; the model learned glaucomatous features from heterogeneous data. The vgg19_bn attained a high degree of AUROC of 99.2%, which was exceeded by ophthalmologists (82.0) and deep-learning systems (97.0) [39], demonstrating its potential for practical use in glaucoma screening. Li et al. trained and validated their model on 31,745 and 8000 fundus images, respectively [40]. The model performed exceptionally well, achieving an AUC of 0.986 with a sensitivity of 95.6%, a specificity of 92.0% and an accuracy of 92.9% for identifying referable glaucomatous optic neuropathy. Our model maintained balance across all performance metrics, as revealed in Table 2, for both glaucoma and healthy cases. The model was unbiased towards any particular class, making it a reliable tool for glaucoma screening for wider populations of glaucoma.

Furthermore, we implemented the DenseNet201, ResNet101 and DenseNet161 architectures (eTable 2 in *Supplementary*). The DenseNet201 demonstrated a classification accuracy of 96%, with an AUROC of 99%. Steen et al. employed the same DenseNet201 architecture, but their model achieved an accuracy of 87.60%, precision of 87.55%, recall of 87.60% and an F1 score of 87.57% on publicly available datasets containing 7299 non-glaucoma and 4617 glaucoma images [41]. Our study has a unique strength in balancing sensitivity and specificity, evident from our model’s high AUROC values—a significant advantage in real-world clinical settings. Many previous models had difficulty maintaining this balance, resulting in high false positive or false negative rates [16, 17, 42].

Liu et al. trained a CNN algorithm for automatically detecting glaucomatous optic neuropathy using a massive dataset of 241,032 images from 68,013 patients, and the model’s performance was impressive [43]. However, the model struggled with multiethnic data (7877) and images of varying quality (884), revealing a drop in AUC with 7.3% and 17.3%, respectively. In contrast, our model demonstrated a modest decline in accuracy, ~9.6%, when tested on the DRISHTI-GS dataset. We suspect that part of this performance shift might be due to inconsistencies and the lack of a clearly defined protocol for glaucoma classification across the publicly available dataset. We discovered specific variances in the classification criteria for glaucoma within the dataset (Fig. 2), which may have contributed to the slight drop in accuracy. Despite this, the model’s accuracy remained potent, indicating a strong generalisation capability. Nevertheless, it would be useful to evaluate the model’s performance across different datasets to confirm its reliability and generality further.

Investigating our model’s top losses, we ascertained two significant insights. First, the model did not perform well in classifying borderline cases, suggesting a need for advanced training techniques to handle such intricacies. Second, we identified potential mislabelling of fundus images within our dataset. This mislabelling could introduce confusion during the model’s learning phase, thereby decreasing performance. Both findings highlight the need for robust data quality checks and expert verification during dataset preparation. To improve the generalisability of the CNN model for glaucoma screening, we should consider accurate data labelled for training a model by glaucoma experts based on clinical tests rather than expanding the fundus data from multiethnic populations. Only five datasets (ACRIMA, REFUGE1, LAG, KEH and PAPILA) classify glaucoma based on comprehensive clinical examinations. In contrast, the remaining datasets used in this study either lacked detailed information regarding their study-specific classification of glaucomatous fundus images, or the diagnosis was solely based on fundus images. Given that accurate data labelling based on clinical tests is essential for training a robust model, the heterogeneity of disease classification may have impacted our model. Additional work profiling the validity and accuracy of these datasets is required.

We explored the decision-making process of our deep-learning model employing Grad-CAM to create heatmaps for the input fundus images. Heatmaps generated using Grad-CAM highlighted the regions of the fundus images that the model analysed when determining the presence or absence of glaucoma. Interestingly, the model’s emphasis areas align well with those that ophthalmologists would typically examine, such as the optic disc and cup, strengthening the clinical relevance of our model. These visual insights add a layer of transparency to our deep-learning model and provide a key link between automated classification and clinical understanding. These insights from the Grad-CAM heatmaps will be invaluable in ensuring the model’s decision-making process correlates with the clinical indicators of glaucoma. This can build clinicians’ trust in these algorithms, allowing for wide adoption in clinical practices.

Although our study demonstrates promising results, there are several limitations. First, our dataset had mislabelled fundus images, which could impact our model’s learning process and accuracy. We employed a data-cleaning procedure to address these challenges, removing 1306 images using ImageClassifierCleaner [30]. This process led to a cleaner and more reliable dataset, upon which we re-trained our model. This refinement considerably enhanced the model’s robustness and improved its generalisation ability to unseen data. Second, we observed that class imbalance could reduce the model’s effectiveness; however, we utilised class weight balance techniques to address this. Furthermore, a data augmentation technique was used in the training phase that could be different from the actual clinical images. Next, our Grad-CAM heatmaps indicated that the model occasionally focused on non-relevant regions for classification decisions, implying that the model might be learning from noise or artifacts within the images. Despite this limitation, the heatmaps confirmed that the model based its predictions on clinically interpretable features. Next, a substantial imbalance in the dataset (eTable 3 in *Supplementary*), with Non-Caucasians representing the majority of healthy cases (76.4%) and Caucasians accounting for nearly half of the glaucoma cases (47.4%), while Hispanics are underrepresented. This may impact our model’s performance and generalisability across different populations. Our study also acknowledges the publicly unavailable glaucomatous fundus data from the African continent (eFigure 1 in *Supplementary*) for our model’s training and validation. Incorporating glaucoma datasets from African countries could be highly beneficial to further enhance our model’s generalisability, especially in under-resourced areas. Finally, our model’s external validation was conducted solely on the DRISHTI-GS dataset. Future studies should aim to validate the model across multiple datasets, diverse populations and varied imaging devices to ensure broader applicability. Additionally, our model did not integrate clinical data, such as patients’ glaucoma history or IOP measurements, and visual field data, which could further enhance its predictive capabilities. Despite these limitations, the potential of our refined model for automated glaucoma screening remains significant and provides exciting prospects for future enhancements.

This research used fundus images to develop a robust computer vision model for glaucoma screening. The best-performing model (vgg19_bn) ascertained high values across multiple evaluation metrics for discerning disease status between glaucoma and healthy cohorts. This model offers a fast, cost-effective and highly accurate tool that can assist eye care practitioners in the decision-making process, ultimately improving patient outcomes and reducing the socioeconomic burden of glaucoma. However, the broad applicability of our model still needs to be validated on more ethnically diverse datasets to ensure its reliability and generalisability. Future studies should focus on the collation of images from people of diverse backgrounds.

## Summary

### What was known before

- Glaucoma detection methods are restricted by improved access to comprehensive eye care, particularly in low- and middle-income countries. Current screening methods have a significant false-positive rate, which can cause unnecessary anxiety and burden for patients. Existing studies focus on specific populations or regions with limited data that lack the potential to be a generalisable computer vision model for glaucoma screening.

### What this study adds

- A robust computer vision model for glaucoma screening using fundus images. This study collected data from 20 publicly accessible databases and a potential model was selected from 20 pre-trained architectures. A computer vision model can assist ophthalmologists and optometrists in the decision-making process, improving patient outcomes and reducing socioeconomic burden. Identification of potential inconsistencies among datasets and the need for refining and validating the model on larger, more diverse datasets to ensure reliability and generalisability.

## Supplementary information

Supplementary Material

## Data Availability

All data used in this study are publicly available, with links provided in the supplementary materials.
