# Supplementary material for: A generalised computer vision model for improved glaucoma screening using fundus images
Source: Eye (Lond). 2024 Nov 5;39(1):109–17. doi: 10.1038/s41433-024-03388-4 (PMC11732976; doi:10.1038/s41433-024-03388-4)
Supplement: Supplementary file 1 — Supplementary Material [file 41433_2024_3388_MOESM1_ESM.pdf]

# Supplementary Figures and Tables:

# A generalised computer vision model for improved glaucoma screening using fundus images

Abadh K Chaurasia,[1*] Guei-Sheung Liu,[1,2,3] Connor J Greatbatch,[1] Puya Gharahkhani,[4,5,6] Jamie E Craig,[7] David A Mackey,[8] Stuart MacGregor,[2,4] Alex W Hewitt,[1,2]

**Affiliations:**

1. Menzies Institute for Medical Research, University of Tasmania, Australia.
2. Centre for Eye Research Australia, Royal Victorian Eye and Ear Hospital, East Melbourne, Australia.
3. Ophthalmology, Department of Surgery, University of Melbourne, East Melbourne, Australia
4. QIMR Berghofer Medical Research Institute, Brisbane, Australia.
5. School of Medicine, University of Queensland, Brisbane, Australia.
6. Faculty of Health, School of Biomedical Sciences, Queensland University of Technology, Brisbane, Queensland, Australia.
7. Department of Ophthalmology, Flinders University, Flinders Medical Centre, Bedford Park, Australia.
8. Lions Eye Institute, Centre for Vision Sciences, University of Western Australia, Australia.

***Correspondence:**

Abadh K Chaurasia

Menzies Institute for Medical Research,

University of Tasmania, Australia.

Email: abadh.chaurasia@utas.edu.au

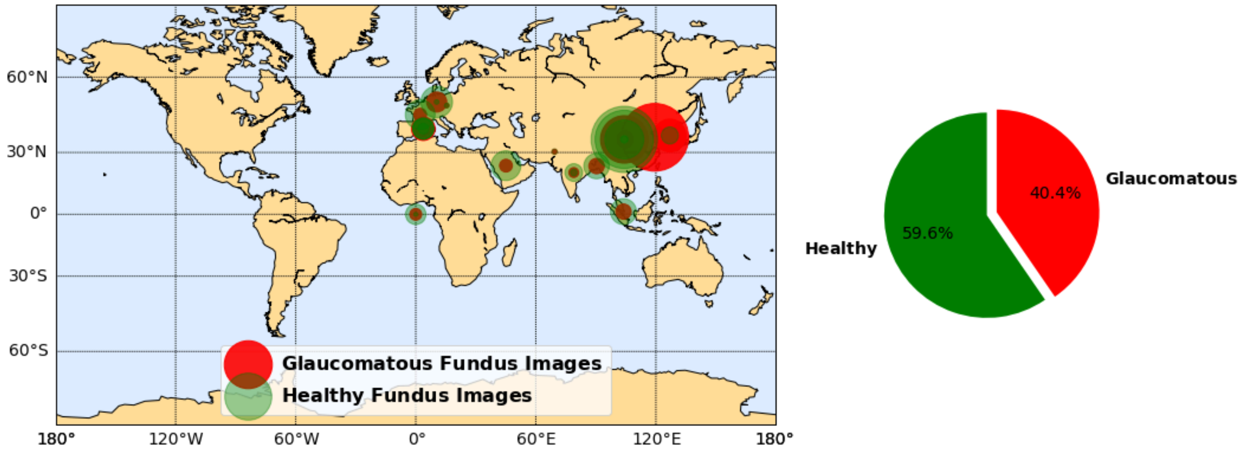


**eFigure 1:** *Highlighting the geographical distribution and comparative proportion of fundus images (glaucomatous and healthy conditions) from publicly accessible databases.*

## SUPPLEMENTARY TEXT

### Training Datasets

#### HRF

The High-Resolution Fundus (HRF) contains 45 disc images: 15 glaucoma, 15 healthy, and 15 diabetic retinopathy. The database was provided by the Pattern Recognition Lab (CS5), the Department of Ophthalmology, Friedrich-Alexander University Erlangen-Nuremberg (Germany), and the Brno University of Technology, Faculty of Electrical Engineering and Communication, Department of Biomedical Engineering, Brno (Czech Republic). The Canon CR-1 fundus camera was used to capture fundus images, with a field of view of 45° from 18 participants. A group made the ground truth of the dataset from experts working in retinal-image analysis and clinicians from the cooperated ophthalmology clinics.

#### ACRIMA

A total of 705 fundus images (396 glaucomatous; 309 normal) were retrieved. The eyes were dilated and centred in the optic disc using the Topcon TRC retinal camera and IMAGEnet® capture System with a field of view of 35°. Two glaucoma specialists with 8 years' experience annotated all the images based on clinical findings. The images of this database come from the ACRIMA project (TIN2013-46751-R) founded by the Ministerio de Economía y Competitividad of Spain.[1]

#### REFUGE1

The Retinal Fundus Glaucoma Challenge (REFUGE), held at the 2018 MICCAI conference, sought to establish a standardised evaluation framework for evaluating

glaucoma detection models that utilise fundus images. A total of 1,200 fundus images were collected from a glaucoma clinic based in China, of which 400 images were procured using a Zeiss VISUCAM with a resolution of 2124 × 2056 pixels and the remaining 800 with a Canon CR-2 with a resolution of 1634 × 1634 pixels. These images were focused on the macula at a 45-degree angle. The reference standard for glaucoma classification was obtained from clinical diagnosis outcomes; it included reviewing IOP measurements, OCT scans, visual field tests, and follow-up examinations. The dataset contains 120 glaucoma cases, either primary open-angle glaucoma or normal-tension glaucoma.

**RIGA**

The retinal fundus images for the glaucoma analysis (RIGA) dataset contains 750 fundus images with OD and OC segmentation ground truth that were manually marked and annotated by six experienced ophthalmologists independently. The fundus images were extracted from three diverse sources: MESSIDOR (460 images), Bin Rushed Ophthalmic Center (195 images), and Magrabi Eye Center (95 images), but these images were ungraded as healthy and glaucoma. A proficient ophthalmologist (AWH) reviewed healthy and glaucomatous images graded by an optometrist (AKC) based on the clinical features of glaucomatous ONH.

**RIM-ONE-DL**

This dataset was a revised version of the three previous versions of RIM-ONE ("v1", "v2", or "v3"); it removed duplicate images and retested images from the earlier data releases, resulting in a unique image per patient. The dataset comprises 313 images

from healthy participants and 172 images from patients with glaucoma. For standardised labelling, two experts reviewed and re-labelled the images, with a third expert consulted in cases of disagreement.[2]

**sjchoi86-HRF**

This dataset included 601 fundus images, which were categorised into four groups: normal (300 images), glaucoma (101 images), cataract (100 images), and retina disease (100 images). The dataset regarding the country of origin, institutes, and clinical information was undisclosed.

**DRIONS-DB**

The Digital Retinal Images for Optic Nerve Segmentation Database (DRIONS-DB) was a collection of 110 coloured digital retinal images gathered from the ophthalmology service at Miguel Servet Hospital in Saragossa, Spain, with detailed annotations provided by two professionals with expertise in ophthalmology. The mean age of the patients was 53.0 years (standard deviation, 13.05), with 46.2% male and 53.8% female, all of Caucasian ethnicity—23.1% of patients had chronic glaucoma, and 76.9% had ocular hypertension. The images were acquired with a colour analogical fundus camera, approximately centred on the ONH, and stored in slide format. The images were scanned using an HP-PhotoSmart-S20 high-resolution scanner in RGB format at a resolution of 600 x 400 and 8 bits per pixel to convert them to a digital format. This dataset was classified as healthy or glaucomatous discs by an optometrist (AKC) based on ONH characteristics and was reviewed by a skilled ophthalmologist (AWH).

## ODIR

The Ocular Disease Intelligent Recognition (ODIR) dataset, aggregated by Shanggong Medical Technology Co., Ltd., incorporates diverse fundus images from different hospitals and medical centres in China. These images, captured using various devices such as Canon, Zeiss, and Kowa, present varied resolutions, contributing to the comprehensive nature of the dataset. The dataset was extensive, comprising 10,000 images from 5,000 patients, into eight distinct categories: normal (N), diabetes (D), glaucoma (G), cataract (C), age-related macular degeneration (AMD - A), hypertension (H), myopia (M), and other diseases/abnormalities (O). The dataset reflects a wide age range for patients with glaucoma, from 24 to 91 years. Professionals labelled the images for the different diseases.

## ORIGA

The Online Retinal Fundus Image Database for Glaucoma Analysis and Research (ORIGA) obtained 650 retinal images from the Singapore Malay Eye Study (SiMES). SiMES was conducted between 2004 and 2007 and represents a comprehensive population-based study targeting adults aged 40 to 80 years.[3] Trained professionals from the Singapore Eye Research Institute properly annotated these retinal images.

## LAG

The Large-scale Attention-based Glaucoma Detection Database (LAG) consists of 4,854 fundus images accessed from the Chinese Glaucoma Study Alliance (CGSA) and Beijing Tongren Hospital. The images of the ONH were taken at various angles using different fundus cameras (Topcon, Canon, and Carl Zeiss). Glaucoma diagnosis in the

dataset was established by qualified specialists using IOP measurements, visual field exams, and manual assessment of the ONH. Approximately 35% of the images were diagnosed as glaucoma cases.

**JSIEC**

The Joint Shantou International Eye Centre (JSIEC) in Shantou, Guangdong province, China, provided 1000 fundus images across 39 different classes out of 209,494.[4] These images were captured using two specific devices: the ZEISS FF450 Plus IR Fundus Camera and the Topcon TRC-50DX Mydriatic Retinal Camera. The images were taken with a field setting of 35-50 degrees.

**BIOMISA**

The Biomedical Image and Signal Analysis (BIOMISA) Research Lab at the National University of Sciences & Technology, Islamabad, Pakistan, collected healthy and glaucomatous fundus and OCT images from 26 subjects. A team of four ophthalmologists annotated these images for the cup-to-disc ratio.[5] The glaucoma-suspected fundus images were excluded from this study.

**BEH**

The dataset consists of coloured fundus images taken with a Topcon Retinal Camera TRC-50DX at the Bangladesh Eye Hospital (BEH) in Dhaka, Bangladesh. The retina images were captured over 2 years, from 2019 to 2020, obtained from Bangladeshi patients aged between 35 to 80 years. The ONH was examined by a pediatric ophthalmologist and a glaucoma surgeon. The dataset contains 463 normal and 171 glaucomatous fundus images.

**VEIRC**

The dataset was extracted from the Venu Eye Institute and Research Centre (VEIRC) in New Delhi, India. The experimental fundus images were acquired from patients aged between 18 to 75 years, using a Welch Allyn Pan Optic Ophthalmoscope with a resolution of 2544 × 1696 pixels. The database contains three categories of images: glaucoma (32 images), suspect glaucoma (107 images), and normal (225 images). Ophthalmologists provided the ground truth of the images.

**LES-AV**

This dataset includes 22 fundus images from different patients with varying resolutions based on the field of view 30 degrees with a resolution of 1444 x 1620 pixels for 21 images and one image with a field of view 45° with a resolution of 1958x2196 pixels.[6] This dataset contains an equal number of glaucomatous and healthy fundus images.

**G1020**

The images of G1020 were taken from a private clinic in Kaiserslautern, Germany, between 2005 and 2017. The images were captured with a 45-degree field of view with dilated pupils, with resolutions of 1944x2108 and 2426x3007 pixels.[7] The standard guidelines often used in routine ophthalmology practice were utilised to form the dataset, consisting of 1020 images sourced from 432 patients—296 images were identified as glaucoma, while the remaining 724 images from 322 patients were classified as healthy.

**PAPILA**

The PAPILA dataset (488 fundus images from 244 people) was collected at the Department of Ophthalmology of the Reina Sofía General University Hospital (HGURS) in Murcia, Spain, between 2018 and 2020. The ophthalmologist or technicians at the HGURS obtained these images using a non-mydriatic Topcon TRC-NW400 device with a resolution of $2576 \times 1934$ pixels.[8] Ophthalmologists graded the patients' data into three classes: glaucomatous, non-glaucomatous, and suspect, based on a comprehensive eye examination.

**KEH**

A total of 1,542 photos were obtained, including 786 photos from patients with normal vision and 756 photos from patients with glaucoma (467 advanced stage and 289 early stage). These images were collected from Kim's Eye Hospital using a non-mydriatic auto fundus camera (AFC-330, Nidek, Japan) with a pixel of 800 X 800. Patients with glaucoma were determined based on clinical tests—typical glaucomatous visual field defects and/or bundle defects of Retinal Nerve Fiber Layers (RNFL) on OCT or red-free RNFL photography. An agreement between two specialists determined the classification of early glaucoma and advanced glaucoma.[9]

**EyePACS**

The Rotterdam EyePACS AIROGS dataset, an extensive collection of 113,893 fundus images, represents a diverse population of 60,357 individuals who visited various centres across the EyePACS network in the United States.[10] The mean age of these participants was 57.1 years, with a standard deviation of 10.4 years. The images, assembled into a training set of 101,442 (publically accessible), underwent a meticulous grading process by a team of 20 expert graders. Each image was reviewed by at least

two graders, who demonstrated outstanding proficiency with a minimum sensitivity of 80% and a specificity of 95% in glaucoma detection from fundus photographs. The team defined 'referable glaucoma' using ten distinct structural features or biomarkers indicative of potential glaucomatous damage, such as optic disc changes and likely corresponding visual field defects.

**External Validation**

**Drishti-GS1**

The Drishti-GS dataset was collected and annotated by clinicians at Aravind Eye Hospital, Madurai, India. It includes 101 fundus images (70 images of glaucoma and 31 normal images) with a field-of-view of 30 degrees with a resolution of 2047×1760 pixels in PNG format. The participants were between 40 and 80 years old, with an equal distribution of males and females. The ground truth of the dataset was constructed based on the presence of notching in the inferior and superior sectors by four experts with varying clinical experience (3 to 20 years). The fundus image was evaluated by a panel of experts who reached a consensus based on the opinions of three out of four participants, determining whether the image was normal or glaucomatous—the selection of patients with glaucoma was made by clinical investigators based on their findings during clinical visits.

**eTable 1:** *The augmentation strategy was applied to broaden and diversify the training dataset.*

| Augmentation Strategy | Description |
|---|---|
| Image Resizing | All images were resized to 512 x 512 pixels and further reduced to 224 x 224 pixels during batch transformations |
| Image Flipping | Both horizontal (do_flip = True) and vertical flipping (flip_vert = True) were performed on the images |
| Rotation | Images were subjected to random rotations up to a maximum of 15 degrees (max_rotate = 15) |
| Image Scaling | Image size scaled randomly, with the smallest scale set to 75% of the original size (min_scale = 0.75) |
| Lighting | Lighting condition variations simulated with a maximum lighting change of 5% (max_lighting = 0.05) |
| Warp | Image warping was disabled (max_warp = 0.0) |
| Affine Transformations | Random affine transformations performed with a probability of 0.8 (p_affine = 0.8) |
| Zoom | Random zoom was applied to the images with a maximum zoom of 10% (max_zoom = 0.1) |

| | |
|---|---|
| Image Normalization | Images were normalised using the statistics of the ImageNet dataset (Normalize.from_stats (*imagenet_stats)) |

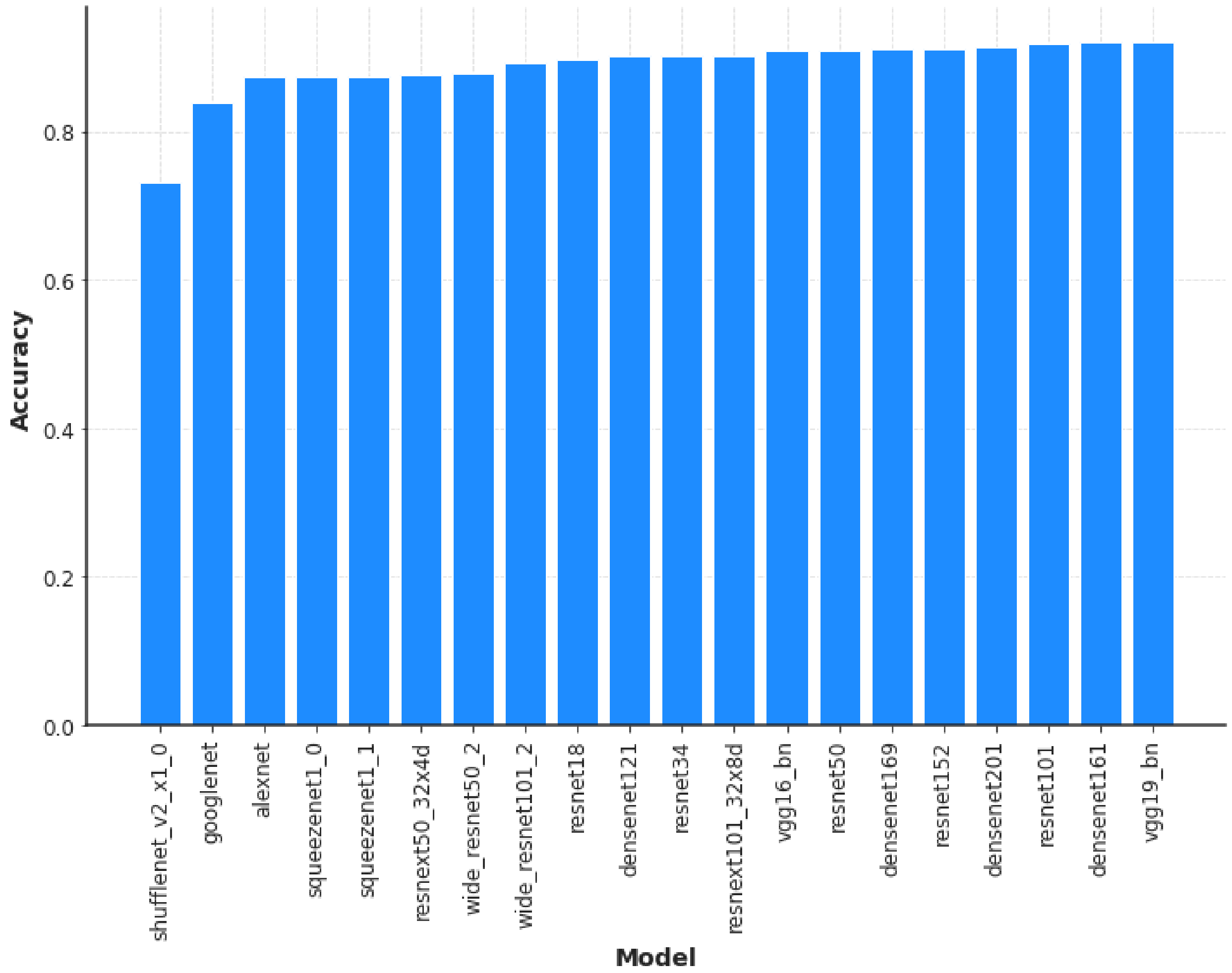


**eFigure 2:** *The performance of pre-trained models was compared on a dataset of 7498 glaucoma and 10869 healthy fundus images.*

**eTable 2:** *Comparative assessment of convolutional neural network-based models for glaucoma screening using multiple classification metrics.*

| Model | Class (Healthy= 2047) Glaucoma =1364) | AUROC | Accuracy | Sensitivity (Recall) | Specificity | Precision | F1-score |
|---|---|---|---|---|---|---|---|
| **vgg19_bn** | Glaucoma | 0.9920 | 0.9671 | 0.9530 | 0.9768 | 0.9654 | 0.9592 |
| | Healthy | 0.9920 | 0.9671 | 0.9768 | 0.9530 | 0.9683 | 0.9725 |
| **resnet101** | Glaucoma | 0.9917 | 0.9654 | 0.9659 | 0.9651 | 0.9459 | 0.9558 |
| | Healthy | 0.9916 | 0.9654 | 0.9651 | 0.9658 | 0.9782 | 0.9716 |
| **densenet161** | Glaucoma | 0.9920 | 0.9639 | 0.9514 | 0.9726 | 0.9605 | 0.9560 |
| | Healthy | 0.9920 | 0.9640 | 0.9726 | 0.9514 | 0.9663 | 0.9694 |
| **densenet201** | Glaucoma | 0.9909 | 0.9578 | 0.9546 | 0.9600 | 0.9406 | 0.9476 |
| | Healthy | 0.9909 | 0.9579 | 0.9600 | 0.9544 | 0.9696 | 0.9648 |

**eTable 3:** *The distribution of glaucoma and healthy cases across different ethnicities based on the predominant population.*

| Group | Countries | Glaucoma (%) | Healthy (%) |
|---|---|---|---|
| Hispanics | Spain | 671 (8.9) | 1050 (9.6) |
| Caucasian | Germany, Czech Republic, France, USA | 3590 (47.4) | 1175 (10.8) |
| Non-Caucasian | China, Saudi Arabia, Pakistan, Bangladesh, India, Singapore, Korea | 3195 (42.2) | 8364 (76.4) |
| Unknown | Unknown | 112 (1.5) | 311 (2.9) |

## Appendix

### Data availability

All the datasets used in this study were obtained from publicly accessible repositories. The links to access the data are provided below:

| Name_of_dataset | Links |
|---|---|
| High-Resolution Fundus Segmentation (HRF) | https://www5.cs.fau.de/research/data/fundus-images/ |
| ACRIMA | https://figshare.com/s/c2d31f850af14c5b5232 |
| Drishti-GS1 | http://cvit.iiit.ac.in/projects/mip/drishti-gs/mip-dataset2/Home.php |
| Retinal Fundus Glaucoma Challenge (REFUGE1) | https://ai.baidu.com/broad/download?dataset=gon |
| Retinal fundus Images for Glaucoma Analysis (RIGA) | https://deepblue.lib.umich.edu/data/concern/data_sets/3b591905z?locale=en |
| RIM-ONE Version 2 DL | http://medimrg.webs.ull.es/ |
| Retina | https://www.kaggle.com/jr2ngb/cataractdataset (https://github.com/yiweichen04/retina_dataset) |

| | |
|---|---|
| Digital Retinal Images for Optic Nerve Segmentation (DRIONS-DB) | http://www.ia.uned.es/~ejcarmona/DRIONS-DB.html |
| Ocular Disease Intelligent Recognition (ODIR) | https://odir2019.grand-challenge.org/Download/ |
| Online Retinal Fundus Image Dataset for Glaucoma Analysis and Research—650 (ORIGA) | https://drive.google.com/drive/folders/1VPCvVsPgrfPNIl932xgU3XC_WFLUsXJR<br><br>https://ieeexplore.ieee.org/abstract/document/5626137<br><br>https://www.kaggle.com/sshikamaru/glaucoma-detection |
| Large-scale Attention-based Glaucoma | https://github.com/smilell/AG-CNN |
| Joint Shantou International Eye Centre (JSIEC) | https://www.kaggle.com/linchundan/fundusimage1000 |
| Mendeley (BIOMISA) | https://data.mendeley.com/datasets/2rnnz5nz74/2 |
| BEH | https://github.com/mirtanvirislam/Deep-Learning-Based-Glaucoma-Detection-with-Cropped-Optic-Cup-and-Disc-and-Blood-Vessel-Segmentation/tree/master/Dataset |
| Harvard Dataverse | https://dataverse.harvard.edu/file.xhtml?persistentId=doi:10.7910/DVN/1YRRAC/OGRSQO&version=1.0 |

| | |
|---|---|
| VEIRC | https://github.com/ProfMKD/Glaucoma-dataset |
| PAPILA | https://figshare.com/articles/dataset/PAPILA/14798004/1 |
| G1020 | https://www.kaggle.com/datasets/arnavjain1/glaucoma-datasets?resource=download |
| LES-AV | https://figshare.com/articles/dataset/LES-AV_dataset/11857698/1 |
| EyePACS-AIROGS | https://airogs.grand-challenge.org/data-and-challenge/ |

## SUPPLEMENTARY REFERENCES


1. Diaz-Pinto A, Morales S, Naranjo V, et al. CNNs for automatic glaucoma assessment using fundus images: an extensive validation. *Biomed Eng Online*; 18. Epub ahead of print 20 March 2019. DOI: 10.1186/s12938-019-0649-y.

2. Batista FJF, Diaz-Aleman T, Sigut J, et al. RIM-ONE DL: A Unified Retinal Image Database for Assessing Glaucoma Using Deep Learning. *Image Anal Stereol* 2020; 39: 161–167.

3. Zhang Z, Yin FS, Liu J, et al. ORIGA-light: An online retinal fundus image database for glaucoma analysis and research, https://ieeexplore.ieee.org/abstract/document/5626137 (accessed 19 July 2023).

4. Cen LP, Ji J, Lin JW, et al. Automatic detection of 39 fundus diseases and conditions in retinal photographs using deep neural networks. *Nat Commun*; 12. Epub ahead of print 10 August 2021. DOI: 10.1038/s41467-021-25138-w.

5. Data on OCT and fundus images for the detection of glaucoma. *Data in Brief* 2020; 29: 105342.

6. Orlando JI, Breda JB, van Keer K, et al. Towards a glaucoma risk index based on simulated hemodynamics from fundus images, http://arxiv.org/abs/1805.10273 (2018, accessed 19 July 2023).

7. Bajwa MN, Singh GAP, Neumeier W, et al. G1020: A Benchmark Retinal Fundus Image Dataset for Computer-Aided Glaucoma Detection, http://arxiv.org/abs/2006.09158 (2020, accessed 19 July 2023).

8. Kovalyk O, Morales-Sánchez J, Verdú-Monedero R, et al. PAPILA: Dataset with fundus images and clinical data of both eyes of the same patient for glaucoma assessment. *Scientific Data* 2022; 9: 1–12.

9. Ahn JM, Kim S, Ahn KS, et al. A deep learning model for the detection of both advanced and early glaucoma using fundus photography. *PLoS One*; 13. Epub ahead of print 27 November 2018. DOI: 10.1371/journal.pone.0207982.

10. Lemij HG, de Vente C, Sánchez CI, et al. Characteristics of a Large, Labeled Data Set for the Training of Artificial Intelligence for Glaucoma Screening with Fundus Photographs. *Ophthalmology Science*; 3. Epub ahead of print September 2023. DOI: 10.1016/j.xops.2023.100300.
